# Automatic quantification of thermographic images of complex regional pain syndrome using radiomics and deep learning

**DOI:** 10.1097/PR9.0000000000001483

**Published:** 2026-09-10

**Authors:** Eline A. van Lange, Elisabeth J. Bijl, Cecile C. de Vos, Jacob M. Mostert, Frank J.P.M. Huygen, Martijn P.A. Starmans

**Affiliations:** a Department of Anaesthesiology, Center for Pain Medicine, Erasmus MC, Rotterdam, the Netherlands; b Department of Radiology and Nuclear Medicine, Erasmus MC, Rotterdam, the Netherlands

**Keywords:** CRPS, Thermography, Radiomics, Deep learning, Machine learning, Classification, Image processing

## Abstract

**Introduction:**

Complex regional pain syndrome (CRPS) is a complication after trauma or surgery. Skin temperature asymmetry is one of the few symptoms that can be measured, for which thermography can be used. However, the current assessment of thermograms relies on manual interpretation and is subjective.

**Objective:**

The aim of this study was to develop an automatic model for quantitative assessment of CRPS based on thermography by using deep learning and radiomics.

**Methods:**

In this study, 178 thermograms of the extremities of 98 patients with CRPS and 837 thermograms from 56 healthy controls were included. A deep learning model was developed to segment the extremities. From each thermogram, for each extremity, 564 radiomics features were extracted. Based on these features, a classification model was developed using a combination of machine learning approaches and evaluated through a 20x random-split cross-validation. The performance of the classification model was compared with visual scoring of the thermograms by 3 clinicians.

**Results:**

The radiomics classification model statistically significantly outperformed all 3 clinicians (mean area under the curve of 0.93 compared with 0.82, 0.79, and 0.69 [*P* < 0.001]).

**Conclusion:**

Our automatic classification model can distinguish thermograms of patients with CRPS from those of healthy controls with a performance that exceeds that of clinicians, thereby providing a basis for future studies evaluating its role in diagnosis and treatment monitoring.

## Abstract 2

Supplemental Digital Content is Available in the Text.

A machine learning model was developed that uses image features to distinguish successfully between thermographic images of patients with complex regional pain syndrome and healthy control subjects.

## 1. Introduction

Complex regional pain syndrome (CRPS) is a complication after trauma or surgery with a prevalence of 21 to 26 per 100 000 people.^23,31^ CRPS is characterized by disproportionate pain in relation to the initial trauma and usually affects distal extremities.^15,40^ The pain in CRPS is accompanied by abnormal sensory, vasomotor, sudomotor, motor, and/or trophic changes.^15^ Complex regional pain syndrome is a clinical diagnosis made using the “New International Association for the Study of Pain (IASP) criteria,”^15^ which are mainly based on the aforementioned clinical symptoms and signs.^2,4^

The pathophysiology of CRPS is still not completely understood, and effective treatment options are limited, which can be attributed to 2 factors. First, the pathophysiology of CRPS is complex and multifactorial. A theory that is gaining increasing support is that autoinflammation plays an important role, particularly in the early stages of CRPS. In the subacute and chronic phases, the harmful effects of autoinflammation, such as neuroplastic and vascular changes, usually play a predominant role.^5,21–23^ The clinical presentation varies between patients and within patients over time, depending on the pathophysiological mechanism that is dominant.^16^ Second, research on CRPS is hampered by the lack of a quantitative, objective method to select eligible patients for appropriate treatment and to assess the effects of interventions.

Both inflammation and vasomotor disturbance cause temperature asymmetry between the affected and contralateral extremity. In the acute phase, the affected extremity is warmer due to inflammation^3^ and reduction of the central sympathetic activity.^42^ In the chronic phase, the affected extremity is colder due to upregulation of alpha-adrenoceptors combined with normalization of central sympathetic activity and endothelial dysfunction, which decreases blood flow.^19^

Thermography is a simple, noninvasive method that detects skin temperature by capturing infrared radiation emitted by the skin.^12^ The resulting image showing the temperature distribution is an intuitive way for clinicians to detect skin temperature patterns, such as asymmetry between affected and unaffected extremity, to support CRPS diagnosis.^9,17,18,26,27,32^ Currently, thermogram assessments are manual and subjective, relying on qualitative evaluation of left-right temperature asymmetry rather than quantitative measurement. Meanwhile, vasomotor disturbances and inflammation can lead to subtle irregular patterns that deviate from normal or vascular occlusion patterns. These patterns are sometimes difficult to distinguish visually. Therefore, there is a need for an improved, automatic, quantitative assessment method of CRPS based on thermograms.

Radiomics refers to the extraction of quantitative features from medical images to objectively describe image characteristics that go beyond visual assessment.^33,37^ Although originating from radiology, many radiomics features are generic and can be applied to thermography, such as measures of temperature distribution and spatial heterogeneity. By quantifying these patterns across the entire extremity, radiomics can provide a more objective and reproducible assessment of inflammation and vasomotor disturbances in CRPS than visual inspection alone.

The aim of this study is to develop a radiomic-based classification model to distinguish thermograms of patients with CRPS from those of healthy controls. This study is a first step toward quantitative thermographic analysis in CRPS.

## 2. Methods

### 2.1. Subjects

Ethical approval for this study was obtained from the Medical Ethics Committee of Erasmus University Medical Center Rotterdam (MEC-2019-0261, MEC-2020-0516). The study protocol conformed to the Declaration of Helsinki 2013.

From patients with CRPS, thermograms acquired during routine clinical care at Erasmus University Medical Center Rotterdam between January 2013 and September 2020 were retrospectively included. Inclusion criteria were age ≥18 years, diagnosis of CRPS according to the New IASP Criteria,^15^ and unilateral CRPS of the distal end of the lower or upper extremity. For patients in which thermograms were acquired at several timepoints due to evaluation of treatment or an aggravation of symptoms, only the thermograms before treatment were included to prevent potential treatment bias. When multiple thermograms were acquired at this timepoint, all were included.

Healthy controls were included prospectively and recruited among hospital employees, medical students, or companions of patients from June 2019 till November 2020. They were ≥18 years and excluded if CRPS had ever been considered in their medical history, or if they had a disease or disability that could influence peripheral blood flow, such as peripheral vascular diseases or diabetes mellitus.

### 2.2. Data collection

All thermograms were made with a Thermacam SC5000 camera (FLIR Systems, Wilsonville, OR) coupled with the associated software package (Altair, version 5.91.010, FLIR Systems). Emissivity was set at 0.99 for skin measurements.^30^ A standardized protocol was developed for thermogram acquisition in healthy controls. Thermograms were acquired of the dorsal side of the lower extremities and both the dorsal and palmar sides of the upper extremities. Since October 2018, this protocol has been implemented in standard clinical care in our hospital.

Approximately 20% of patients with CRPS suffer from dystonia,^14,40^ a fixed flexion posture, which may also be observed in thermograms. To prevent a potential bias due to dystonia, additional thermograms were acquired of healthy controls simulating common stand deviations. For the classification model development, the dataset of the healthy control group was supplemented with these thermograms of postural abnormalities until the same percentage was achieved as in the dataset of patients with CRPS. In addition, several thermograms with artefacts, such as wearing rings, were acquired intentionally to create a dataset comparable to that of the patients with CRPS.

### 2.3. Segmentation using deep learning

In radiomics, features are extracted from a region of interest, which requires segmentation of the thermogram. In our study, we focused on both the affected and the contralateral extremity. As manual segmentation is time-consuming and not feasible in daily clinical practice, we implemented automatic segmentation using deep learning, specifically a U-Net convolutional neural network.^1,29^ The U-Net is specifically designed for medical image segmentation and enables consistent extraction of regions of interest for radiomics analysis.

For development and validation of the segmentation model, ground truth segmentations were created by semi-automatically segmenting the extremities from the background using in-house software,^35^ followed by manual refinement. The U-Net segmentation model was evaluated in a 20x random-split cross-validation^24,28^ using the dice similarity coefficient (DSC) as loss function. In each iteration, the data were randomly split into 80% of the subjects for training and 20% for testing in a stratified manner, to make sure the class distribution in all sets was similar to that in the full dataset. In this way, thermograms of the same subject were grouped together. The obtained segmentations from the U-Net were automatically split into separate segmentations of the left and right extremities through connected component analysis.

On the test datasets in the cross-validation, the segmentation model had an almost perfect performance with a mean DSC of 0.99 (95% CI: 0.99-0.99). There was no substantial performance difference between patients with CRPS (DSC: 0.99 [95% CI: 0.98-0.99]) and healthy controls (DSC: 1.00 [95% CI: 0.99-1.00]), nor between upper extremities (0.99 [95% CI: 0.99-1.00]) and lower extremities (0.99 [95% CI: 0.98-0.99]).

Given its robust performance, the automatic segmentation model was integrated into the classification model. To generate automatic segmentations for all thermograms used by the classification model, the U-Net was reevaluated in a 10-fold cross-validation such that for every thermogram an automatic segmentation was made.

### 2.4. Classification using radiomics

For the classification model, all acquired thermograms were visually inspected for artefacts or acquisition protocol discrepancies. Thermograms were excluded if they contained only one hand or foot, if the 2 hands or feet were visible as one object, or if a part of an extremity was missing. Thermograms with only toe or finger tips missing were included.

**Figure 1.**
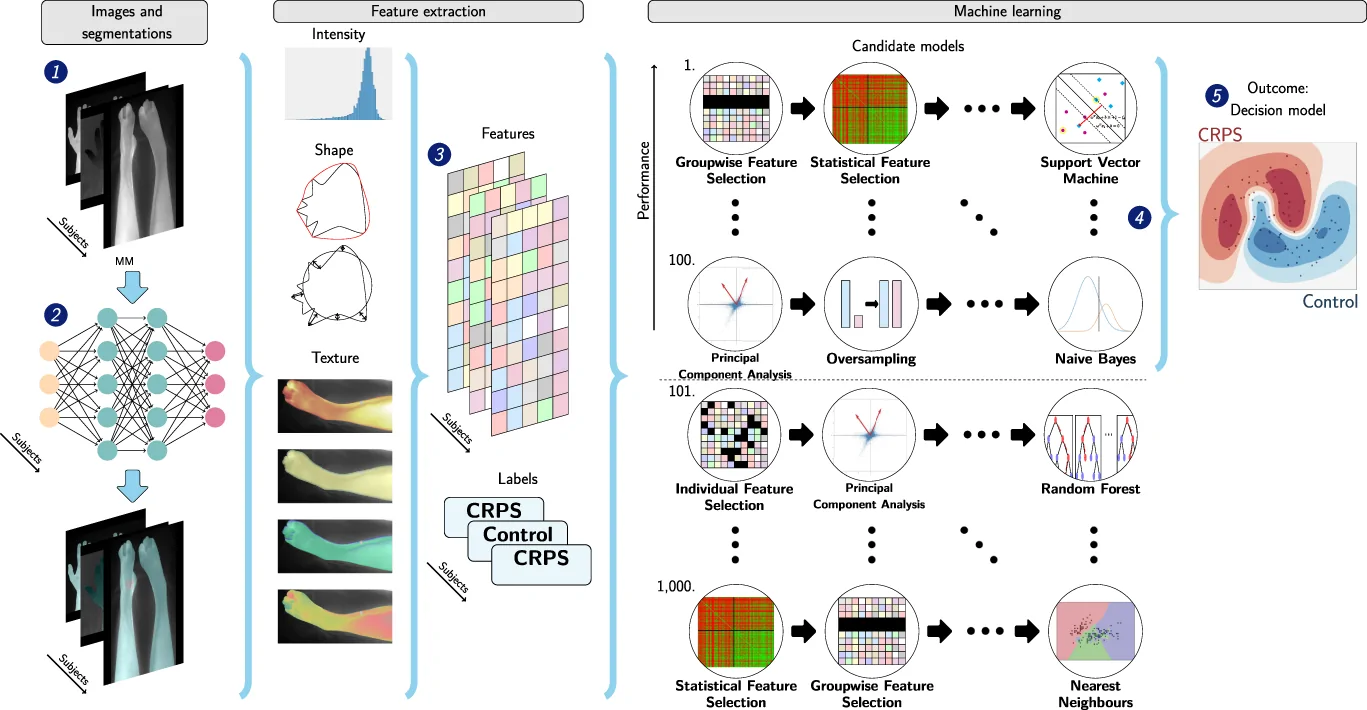
Schematic overview of the automatic thermography quantification model development. The thermograms (1) are first processed through a convolutional neural network (2) to segment the extremities from the background. Processing steps include feature extraction (3) and the creation of a machine learning decision model (5), using an ensemble of the best 100 workflows from 1,000 candidate workflows (4), where the workflows are different combinations of the different analysis steps (eg, the classifier used). Adapted from Reference 41: the scans have been adopted to this study and the convolutional neural network has been added.

An overview of the radiomics methodology is depicted in Figure 1. To extract radiomics features and create a decision model based on these features, the Workflow for Optimal Radiomics Classification (WORC) framework was used.^36,38^ Workflow for Optimal Radiomics Classification is an automated machine learning framework for radiomics in which the creation of a decision model consists of several steps, ie, feature selection, resampling, and machine learning. For each thermogram, 564 default WORC features were extracted per segmented extremity, resulting in a total of 1128 features. These features quantify intensity (13 features, eg, minimum temperature, maximum temperature, temperature distribution), shape (43 features, eg, compactness, sphericity), and texture (508 features, eg, heterogeneity, gradient). Based on these features, WORC performs an automated search across 1,000 candidate workflows, ie, specific combinations of algorithms for each step and their associated parameters, to maximize classification performance on the training set. The resulting decision model consists of an ensemble of the 100 best workflows by averaging the posterior probabilities to create a single classification per thermogram.

The radiomics method was evaluated using the same 20x random-split cross-validation as the segmentation U-Net. All model optimization was performed within the training set using an internal 5x random-split cross-validation to minimize risk of overfitting on the test set.^38^

Several classification models were created: Model based on (1) the automatic segmentations (the CRPS classification model); (2) manual segmentations; (3) intensity features only; (4) shape features only; (5) texture features only; (6) an age-matched subset; (7) hands only; and (8) feet only. Model 2 was created to verify that the CRPS classification model performed similarly on the automatic and the manual segmentations. In all other models, the automatic segmentations were used to extract the radiomics features. Models 3, 4, and 5 were created to assess which type of features were most distinctive for classifying CRPS. Model 6 was created to assess a potential age bias. Models 7 and 8 were created to assess whether one model combining hands and feet would perform equally well as separate models for hands and feet.

In addition, to enable a more direct comparison with previous thermography studies,^6–10^ temperature asymmetry was also evaluated as the absolute difference in mean temperature between the extremities for each thermogram. For simplicity, the performance was computed using the entire dataset.

### 2.5. Classification by clinicians

To compare the CRPS classification model with clinical practice, a set of thermograms was visually scored by 3 clinicians (Clin1: 4 years, Clin2: 3 years, and Clin3: 5 years of experience). The set was created by randomly selecting one thermogram per subject. The clinicians were blinded to the diagnosis, but aware of the inclusion and exclusion criteria. The classification was done using a four-point scale to indicate the certainty of the clinicians: 1 = CRPS, certain; 2 = CRPS, uncertain; 3 = control, uncertain; and 4 = control, certain. For binarization of the clinicians' scores, scores of 1 and 2 were converted to CRPS, and 3 and 4 were converted to control.

### 2.6. Statistical analysis

To evaluate the difference in the demographic characteristics and the classification value of the individual radiomics features between the patients with CRPS and healthy controls, univariate statistical testing was performed using a Mann–Whitney *U* test for continuous variables and a χ^2^ test for categorical variables. *P*-values of the demographic characteristics were not corrected for multiple testing as these are purely descriptive: *P*-values of the radiomics features were corrected using the Bonferroni correction, ie, multiplying the *P*-value with the number of tests conducted. For all statistical tests, *P* ≤ 0.05 was considered statistically significant.

To evaluate the performance of the classification model and the clinicians, we calculated the area under the curve (AUC) of the receiver operating characteristic (ROC) curve, and the Balanced Classification Rate (BCR),^39^ which is a class-balanced alternative of accuracy, the sensitivity, and the specificity. Receiver operating characteristic confidence bands were constructed using fixed-width bands.^20^ For the BCR, sensitivity, and specificity metrics of the clinicians, the binarized scores were used. The positive class was defined as the patients with CRPS. The 95% confidence intervals of the performance metrics were constructed using the corrected resampled *t* test, thereby taking into account that the samples in the cross-validation splits are not statistically independent.^24^

To enable direct statistical comparison between classification by the clinicians and the CRPS classification model (model 1), this model was additionally evaluated in a leave-one-out cross-validation. The AUCs of the CRPS classification model from this cross-validation and the classification by the clinicians were compared using a DeLong test.^11^ To gain insight into the CRPS classification model's decision making, the subjects were ranked based on the probability between 0% and 100% of being a patient with CRPS as classified by the model. Ranking was done as correctly classified control (ground truth: control, probability: near 0%)—misclassified CRPS (ground truth: CRPS, probability: near 0%)—misclassified control (ground truth: control, probability: near 100%)—correctly classified CRPS (ground truth: CRPS, probability: near 100%). This ranking was compared with a ranking based on the clinicians' binary scores, where thermograms were considered correctly classified if all 3 clinicians were correct and misclassified if all 3 clinicians were incorrect.

## 3. Results

### 3.1. Data

In this study, 98 patients with CRPS and 56 healthy controls were included (Table 1). A statistically significant difference was found in age between patients with CRPS (interquartile range [IQR]: 30-58 years) and healthy controls (IQR: 23-40 years) (*P* = 0.001), but not in gender.

**Table 1:** Demographic and thermographic classification subsets characteristics of patients with complex regional pain syndrome (CRPS) and healthy controls.

	Patients with CRPS (N = 98)	Healthy controls (N = 56)	*P*
Sex, N of females (%)	86 (88%)	49 (88%)	0.963*
Age in years (median [IQR])	48 [30-58]	33 [23-40]	0.001†
Number of thermograms	178	249	
Upper extremities (%)	74 (42%)	161 (65%)	
Lower extremities (%)	104 (58%)	88 (35%)	
Dystonia/simulated stand deviations	45 (25%)	62 (25%)	

* χ^2^ test.

† Mann–Whitney *U* test.

IQR, interquartile range.

The total dataset in this study consisted of 1015 thermograms: 178 of patients with CRPS, 837 of healthy controls, of which 650 were simulated stand deviations. The dataset used for the CRPS classification models consisted of 427 thermograms, including all thermograms of all patients with CRPS and 249 thermograms of all 56 healthy controls, of which 62 were simulated stand deviations (Table 1).

### 3.2. Thermogram deviations

Dystonia was observed in 45 thermograms of patients with CRPS. For the patients with CRPS, majority of thermograms were made before the implementation of a standard acquisition protocol. Therefore, 86% of thermograms of patients with CRPS still showed one or more protocol deviations, whereas only 21% of the thermograms of healthy controls showed protocol deviations. However, several of the most common protocol deviations involve artefacts that do not affect the depiction of the extremity and can be removed by correct segmentation of the thermogram. An overview of the protocol deviations observed in the thermograms is shown in Table 2.

**Table 2:** Frequency of artefacts and protocol deviations in the thermograms used for the classification models of both the patients with complex regional pain syndrome (CRPS) and healthy controls.

No. of thermograms with artefacts or protocol deviations (%)	Thermograms patients with CRPS (N = 178)	Thermograms healthy controls (N = 249)
Object in the background	134 (75%)	57 (23%)
Hot spots around extremities	119 (66%)	14 (6%)
Halo around extremities	3 (2%)	6 (2%)
Finger or toe tips indistinguishable due to cold	61 (34%)	29 (12%)
Missing parts	33 (19%)	6 (2%)
Clothes visible	9 (5%)	17 (7%)
Jewelry	24 (13%)	14 (6%)
Extremities overlap or touching	19 (11%)	1 (0%)
Fingers not well spread (in case of hands)	39 (57%)	11 (8%)

### 3.3. Classification using radiomics

**Figure 2.**
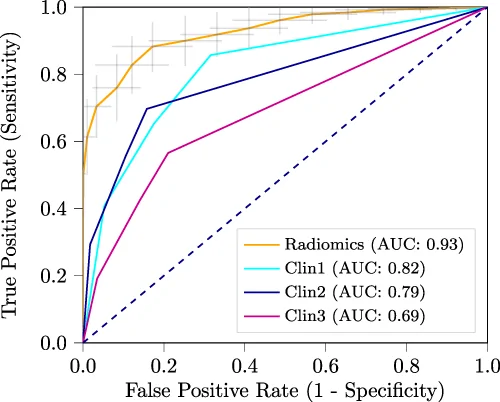
Receiver operating characteristic (ROC) curves of the CRPS classification model (model 1) and the 3 clinicians (Clin1, Clin2, Clin3) to distinguish patients with complex regional pain syndrome (CRPS) from healthy controls. For the classification model, the grey lines identify the 95% confidence intervals of the 20x random-split cross-validation; the orange curve is fit through the means. AUC, area under the curve.

Model 1, the CRPS classification model with automatic segmentation, had a mean AUC of 0.93 (95% CI: 0.90-0.97), indicating high performance in differentiating thermograms of patients with CRPS from healthy controls, and the feasibility of our automatic segmentation method. The performance of model 1 is depicted in Table 3, and the ROC curve is shown in Figure 2.

**Table 3:** Performance of the complex regional pain syndrome classification model (model 1) and the 3 clinicians (Clin1, Clin2, Clin3).

	Radiomics—model 1 Mean [95% CI]	Clin1	Clin2	Clin3
AUC	0.93 [0.90, 0.97]	0.82	0.79	0.69
BCR	0.84 [0.79, 0.88]	0.74	0.73	0.64
Sensitivity	0.75 [0.65, 0.89]	0.65	0.56	0.42
Specificity	0.92 [0.85, 0.98]	0.82	0.89	0.86

AUC, area under the curve; BCR, balanced classification rate; CI, confidence interval.

The performances of the other classification models (models 2–8) are depicted in Supplementary Table S1, http://links.lww.com/PR9/A430, and the ROC curves are shown in Supplementary Figure S1, http://links.lww.com/PR9/A430. When using the manual segmentations (model 2), the performance was similar to model 1, with a mean AUC of 0.94 (95% CI: 0.90-0.97). Upon using only a single type of radiomics features, the performances were similar to that of the model using all features when using either only intensity (model 3: AUC of 0.93 [95% CI: 0.90-0.97]), shape (model 4: AUC of 0.89 [95% CI: 0.85-0.94]), or texture features (model 5: AUC of 0.91 [95% CI: 0.88-0.94]). This indicates that using one of these feature groups may already be sufficient to distinguish thermograms of patients with CRPS from healthy controls. In contrast, temperature asymmetry, defined as the absolute difference in mean temperature between the extremities, showed a substantially lower discriminative performance when evaluating all thermograms (AUC: 0.79). In the age-matched subset, which consisted of 268 thermograms of 56 healthy controls and 48 patients with CRPS, performance was similar (model 6: AUC of 0.93 [95% CI: 0.89-0.96]), indicating that the classification model is age-independent. Finally, the performance when only including thermograms of the hands (model 7: AUC of 0.93 [95% CI: 0.88-0.97]) or feet (model 8: AUC of 0.96 [95% CI: 0.92-0.99]) was similar as well, indicating that differentiating between hands and feet is not necessary when creating a CRPS classification model based on radiomics features.

### 3.4. Comparison with clinicians

The performance of the 3 clinicians scoring each thermogram as patient with CRPS or healthy control is depicted in Table 3; the ROC curves are shown in Figure 2. The AUC of the CRPS classification model (model 1) was statistically significantly better than that of all 3 clinicians (*P* < 0.0001).

**Figure 3.**
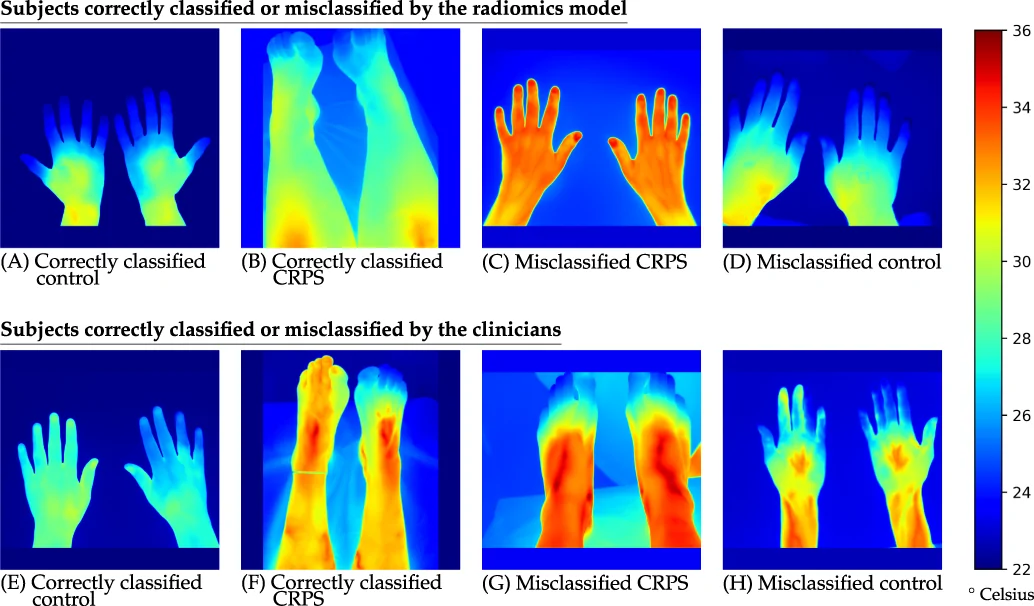
Thermograms of patients with complex regional pain syndrome (CRPS) and healthy controls that are classified correctly or misclassified either by the radiomics model (top row) or all 3 clinicians (bottom row).

The 3 clinicians concordantly correctly classified 34 of the 98 thermograms of patients with CRPS (35%) and 43 of the 56 thermograms of healthy controls (77%). Of these correctly classified thermograms, the CRPS classification model correctly classified 30 CRPS thermograms and 42 control thermograms, thus showing high agreement with the clinicians. All 3 clinicians misclassified the same 29 thermograms of patients with CRPS (30%) and 3 thermograms of healthy controls (5%). Generally, misclassified thermograms for the clinicians were healthy controls who displayed temperature asymmetry or patients with CRPS showing no visually apparent asymmetry, see Figure 3 for several examples. Of the thermograms misclassified by the clinicians, the CRPS classification model correctly classified 20 CRPS thermograms and all control thermograms, showing the potential complementary value of the model as a diagnostic aid for clinicians.

### 3.5. Model insight

In the univariate statistical testing of the individual features, 672 of the total 1128 features (59%) showed statistically significant differences between patients with CRPS and healthy controls. These consist of 18/26 intensity, 8/86 shape, and 646/1016 texture features, corresponding with the observations from the models using only a single group of these features.

The CRPS classification model only classified a small number of thermograms incorrectly. By visually inspecting those borderline thermograms, ie, the thermograms with a probability around 50%, it was observed that they showed a relatively uniform temperature distribution in the extremities. The thermograms that were correctly classified did not show common visual characteristics indicating thermograms of either patients with CRPS or healthy controls. These findings suggest that radiomics classifies the thermograms based on patterns in features which are beyond visual perception, underscoring the potential of thermography to provide more information than can be detected by humans.

## 4. Discussion

In this study, we successfully developed a combined segmentation and classification model, to distinguish thermograms of unilateral patients with CRPS from healthy controls using radiomics and deep learning.

Currently, CRPS diagnosis is mainly based on clinical evaluation. Thermograms can be used by clinicians as a diagnostic aid, because various characteristics such as skin temperature asymmetry can be examined manually by a physician or seen by the human eye on a thermogram.^26^ However, even for clinicians who frequently use thermography, our results indicate that visual assessment of thermograms can be challenging and observer dependent. Our classification model was significantly better at recognizing thermograms of patients with CRPS than 3 clinicians and is observer independent, automatic, and quantitative. Our model could, therefore, significantly increase the value of thermography as a diagnostic aid, especially on cases of CRPS with lack of clinical consensus or with subtle and difficult to detect abnormalities.

Several models were created to assess the influence of segmentation, feature groups, demographics, and affected body part. These assessments verified that the CRPS classification model with automatic segmentation performed comparable to using manual segmentation. Moreover, the classification model performed well regardless of patient age, indicating that the model does not make use of age-related differences and showed similar performance for hands and feet. Future research should evaluate performance in other regions such as knees or lower legs. The intensity, shape, and texture feature groups all included individually significant features, as shown in the univariate analyses. The radiomics models using a single feature group all performed similarly to the model using all features. Although an intensity-only model may be attractive due to fewer features and its interpretability, a broader feature set may be more robust to fluctuations in individual features and better suited to capture heterogeneity within CRPS.

The strong performances of our classification model suggest that thermograms capture complex temperature patterns beyond their primary clinical use of assessing temperature asymmetry. The ability of the model to distinguish this heterogeneous CRPS group from healthy controls indicates that it captures shared thermographic patterns across patients with CRPS, rather than relying on a single characteristic or subtype. Moreover, insight into contributing feature types can be obtained at the group level. Since all patients had unilateral CRPS, our model based solely on the 26 intensity features confirmed that differences in skin temperature distribution between the extremities are a distinguishing factor. Similarly, our model based only on the 646 texture features showed that patients with CRPS not only exhibit temperature differences but also altered spatial patterns. Together, these results highlight the potential of radiomic-based thermographic analysis to identify pathophysiological abnormalities beyond simple temperature asymmetry and provide a basis for future studies relating model predictions to underlying physiological processes to improve interpretability.

In our study, we tried to limit the effects of dystonia, artifacts, and acquisition deviations. Dystonia might have biased the CRPS classification because dystonia is normally only present in patients with CRPS and not in healthy controls. In our study, 25% of patients with CRPS showed dystonia, which is consistent with the incidence of dystonia found in previous research (20%).^14,40^ To avoid bias due to postural deviations resulting from dystonia in patients with CRPS, additional thermograms were taken of healthy controls simulating common stance abnormalities to balance the dataset. Although this approach is not perfect, the strong performance of the CRPS classification model and the completely shape-independent intensity model indicate that stand posture abnormalities did not affect the model. In addition, most of the artefacts and discrepancies in the acquisition protocol were removed by extracting features solely from segmentations of the extremities, since they mostly occur in the image background.

Studies on the use of thermography in CRPS are scarce and mainly focus on temperature asymmetry.^6–10,17,25–27^ These studies are often based on small, manually selected regions of interest, effectively taking point measurements.^6,7,9,10,17,25^ They usually focus on the plantar foot^6–8^ and temperature differences.^7,8,10,25–27^ As a result, findings are influenced by observer-dependent region of interest and CRPS patient variability. Our results indicate that temperature asymmetry, even when computed on fully automated complete extremity segmentations, yields only moderate discriminative performance. In contrast, our contributions include automatic segmentation of the entire CRPS affected area to minimize observer bias, analysis of a large set of image features (using radiomics) to capture variations between different CRPS pathologies, evaluation of a large sample size with various affected extremities to ensure robust evidence, and comparison of model performance with clinical practice. These factors, and the fact that our methods are fully automated and thus observer independent, improve their feasibility to assess treatment effects.

The main limitation of our study is the lack of independent, external validation of our classification model. Due to the sensitivity of thermography to hardware, acquisition protocols, and environmental conditions, generalizability remains unknown and external validation is required. However, we applied rigorous internal cross-validation with 20 training–test splits in a relatively large and heterogeneous cohort. Since all model optimization was restricted to the training set, the risk of overestimating performance on the test set due to “over-engineering” was limited, strengthening the evidence compared with previous CRPS thermography studies.

In the current study, we deliberately compared patients with CRPS with healthy volunteers to identify CRPS-related thermographic abnormalities. The ability of the model to distinguish a heterogeneous CRPS cohort from healthy controls provides a robust, observer-independent reference to normal physiology and a necessary first step toward more clinically specific diagnostic models. Consequently, thermograms outside this context would be classified into one of the 2 categories, precluding differentiation of CRPS from other conditions. Future studies should extend this work by evaluating the performance of radiomics-based thermography in distinguishing CRPS from other post-traumatic or neuropathic pain conditions encountered in clinical practice.

Moreover, CRPS was modeled as a single diagnostic entity in this study. Future studies should investigate whether our model can further differentiate patients with CRPS based on their underlying pathophysiological mechanisms, disease subtypes, and treatment response. Currently, CRPS treatment is symptom-based, but there is increasing support for a more mechanism-based treatment approach.^2,4,5,13,34^ Based on our findings, we hypothesize that radiomics for thermographic analysis could contribute to future effects aimed at distinguishing and better understanding the biological CRPS mechanisms involved. Ultimately, after validation in future studies, this approach could assist clinicians in diagnosing CRPS, stratifying patients by underlying pathophysiology, and monitoring treatment effects.

In conclusion, our radiomics model can automatically distinguish thermograms of patients with CRPS from those of healthy controls, outperforming clinicians in the same task. These findings demonstrate the feasibility of quantitative, observer-independent thermographic analysis in CRPS. Future studies should assess whether this approach can distinguish CRPS from other conditions, and whether it can further differentiate CRPS mechanisms and treatment effects.

## Disclosures

The authors have no conflict of interest to declare.

## Supplemental digital content

Supplemental digital content associated with this article can be found online at http://links.lww.com/PR9/A430.
