# Supplementary material for: Automatic quantification of thermographic images of complex regional pain syndrome using radiomics and deep learning
Source: Pain Rep. 2026 Sep 10;11(5):e1483. doi: 10.1097/PR9.0000000000001483 (PMC13568389; doi:10.1097/PR9.0000000000001483)
Supplement: Supplementary file 1 [file painreports-11-e1483-s001.pdf]

Supplementary Table S1. Performance of the classification models: based on manual segmentation (Model 2); only using intensity features (Model 3); only using shape features (Model 4); only using texture features (Model 5); using an age-matched subset (Model 6); only based on hands (Model 7); and only feet (Model 8). The mean and 95% confidence intervals are reported.

**Model**	**2: Manual**	**3: Intensity**	**4: Shape**	**5: Texture**
**AUC**	0.94 [0.90, 0.97]	0.93 [0.90, 0.97]	0.89 [0.85, 0.94]	0.91 [0.88, 0.94]
**BCR**	0.84 [0.79, 0.90]	0.84 [0.79, 0.89]	0.80 [0.74, 0.86]	0.83 [0.79, 0.87]
**Sensitivity**	0.75. [0.67, 0.84]	0.76 [0.67, 0.85]	0.67 [0.55, 0.79]	0.75 [0.66, 0.85]
**Specificity**	0.93 [0.87, 0.99]	0.92 [0.86, 0.99]	0.93 [0.88, 0.98]	0.90 [0.86, 0.94]
**Model**	**6: Age-matched**	**7: Hands**	**8: Feet**	
**AUC**	0.93 [0.89, 0.96]	0.93 [0.88, 0.97]	0.96 [0.92, 0.99]	
**BCR**	0.82 [0.76, 0.88]	0.80 [0.74, 0.87]	0.89 [0.83, 0.94]	
**Sensitivity**	0.68 [0.57, 0.80]	0.67 [0.54, 0.80]	0.87 [0.78, 0.95]	
**Specificity**	0.96 [0.93, 1.00]	0.94 [0.88, 0.99]	0.91 [0.83, 0.98]	

AUC: area under the curve; BCR: balanced classification rate;

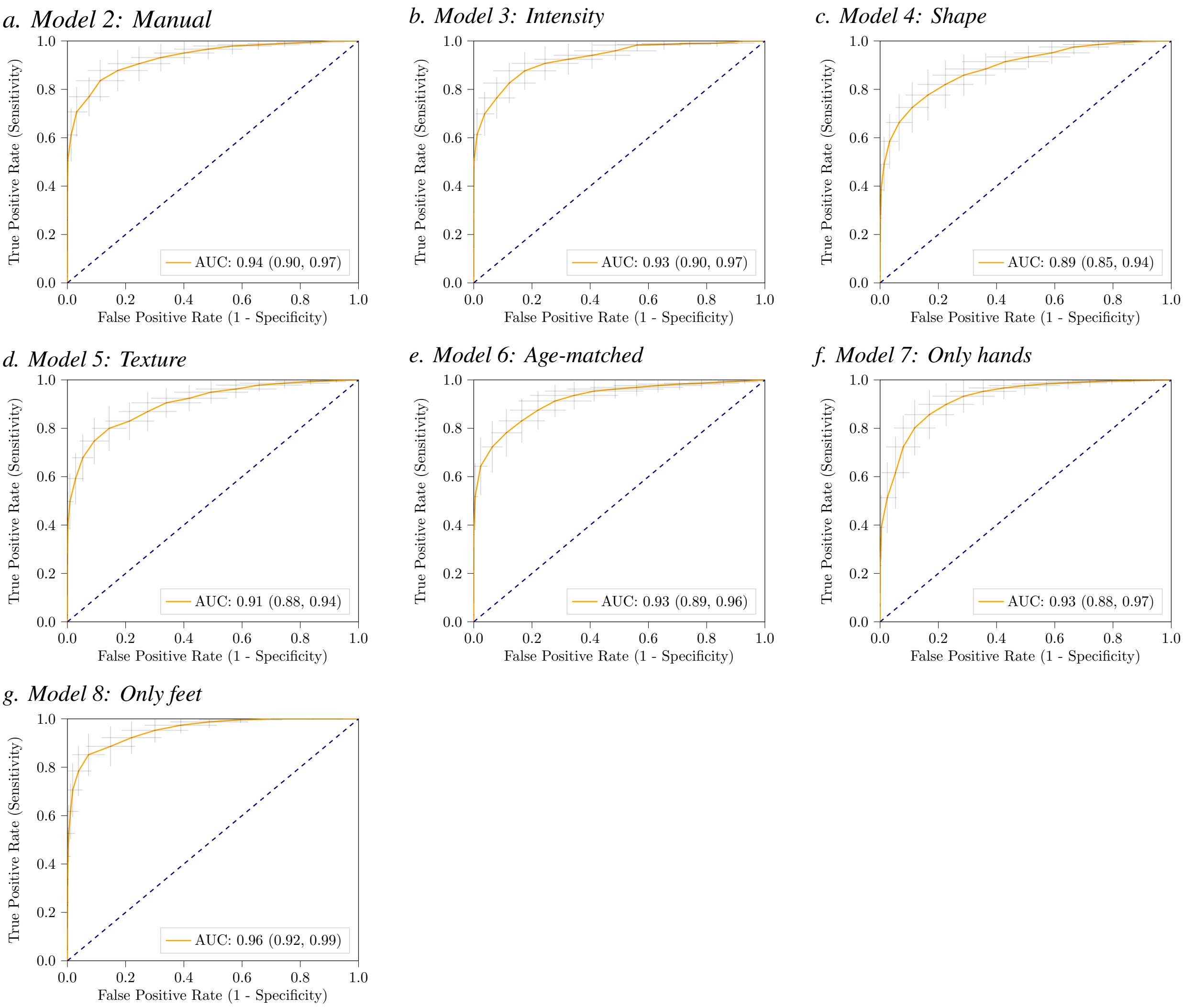

Supplementary Figure S1. Receiver operating characteristic (ROC) curves of seven radiomics models to distinguish patients with complex regional pain syndrome from healthy controls based on: a) manual annotations; b) only intensity-based features; c) only shape-based features; d) only texture-based features; e) an age-matched subset; f) only thermograms of hands; and g) only thermograms of feet. The grey lines identify the 95% confidence intervals of the 20× random-split cross-validation; the bold curves are fit through the means. AUC: area under the curve.
